# Physical Activity, Sedentariness, Eating Behaviour and Well-Being during a COVID-19 Lockdown Period in Greek Adolescents

**DOI:** 10.3390/nu13051449

**Published:** 2021-04-24

**Authors:** Ioannis D. Morres, Evangelos Galanis, Antonis Hatzigeorgiadis, Odysseas Androutsos, Yannis Theodorakis

**Affiliations:** 1 Department of Physical Education and Sport Science, University of Thessaly, 42100 Trikala, Greece; iomorres@pe.uth.gr (I.D.M.); v.galanis@hotmail.com (E.G.); theodorakis@pe.uth.gr (Y.T.); 2 Department of Nutrition and Dietetics, University of Thessaly, 42132 Trikala, Greece; oandroutsos@uth.gr

**Keywords:** pandemic, COVID-19, lockdown, exercise, sport, diet, mood, quality of life

## Abstract

Adolescents’ daily life has dramatically changed during the COVID-19 era due to the social restrictions that have been imposed, including closures of schools, leisure centers and sport facilities. The purpose of this study was to examine levels of well-being and mood and their relations with physical (in)activity and eating behaviors in adolescents during a lockdown period in Greece. A total of 950 adolescents (Mean Age = 14.41 years ± 1.63) participated in a web-based survey while education was conducted online and organized sport activities were interrupted. Participants showed poor well-being, insufficient physical activity levels and moderate scores of healthy eating behavior. Hierarchical regression analysis showed that, after controlling for the effect of gender and body mass index, increased physical activity and healthier eating behavior predicted better well-being (b = 0.24, *p* < 0.01 and b = 0.19, *p* < 0.01, respectively), whereas sedentariness predicted worse well-being (b = −0.16, *p* < 0.01). Furthermore, it was revealed that days of physical activity per week was a stronger predictor of well-being than minutes of physical activity per week, and that both in-house and out-of-house physical activity were beneficial. Considering that well-being in our study was below the threshold recommended by the World Health Organization as indicative of possible depressive symptoms, measures to increase physical activity, decrease sedentariness and improve eating behavior should become a priority for communities and policy makers.

## 1. Introduction

Physical activity is defined as “any bodily movement produced by skeletal muscles that results in energy expenditure” [1] and includes various subsets such as walking, cycling or sport. A subset of physical activity that is planned, structured, repetitive and purposive to fitness gains is typically called ‘‘exercise’’ [1]. Physical activity is a preventive/therapeutic factor towards psychological well-being components or proxies while sedentariness an adverse factor. Particularly, large-scale cross sectional studies in adults have linked physical activity to lower risk of depression [2] and sedentariness to higher risk of depression [3]. Furthermore, systematic reviews or cross sectional studies found that exercise is related with lower depression [4,5] or anxiety [6] in adult samples. Various systematic reviews of cross sectional and empirical studies in young people (<18 years) have also demonstrated the positive and negative role, respectively, of physical activity and sedentariness in well-being [7,8]. Similarly, meta-analyses showed that physical activity in young people improves well-being components such as depression [9,10] or anxiety [11,12]. The World Health Organization (WHO) [13] guidelines for better mental health recommend physical activity of ≥420 min/week for adolescents, a larger amount of time than for adults (150–300 min/week). This highlights the importance of increased physical activity for better well-being in adolescents.

However, physical activity and well-being are decreasing worldwide after the COVID-19 pandemic declaration by the WHO in March 2020. Particularly, lockdown measures via social distance restrictions including closures of schools, leisure facilities and sport clubs have been repeatedly introduced to contain the spread of the SARS-CoV-2 virus but led adolescents to lower physical activity and well-being [14,15,16,17,18,19]. In spite of this rather somber reality, some encouraging findings were provided by a study conducted in China [16], which reported that even limited weekly physical activity (>60 min of weekly) was linked to lower risk of deteriorated well-being in young people during the COVID-19 pandemic. In line, the WHO [13] recommends >60 min/day of moderate-vigorous physical activity for better mental health in adolescents. The importance of physical activity for adolescents is highlighted further by a series of research findings in the era of COVID-19. Particularly, it has been found that physical activity lowers sedentariness and sleep disturbance [19], which are, in turn, linked to an improvement of the widely deteriorated dietary behavior [20,21]. The importance of addressing healthy dietary behavioral patterns should represent a constant concern. State-of-the-art outlines have reported an update for healthy eating across five continents and for Mediterranean diet, with physical activity being an inherent component [22,23].

In the imposed COVID-19 lockdown period in Greece (spring 2020), adults showed decreased physical activity [24] increased depressive symptoms [25], disturbed eating behavioral patterns [26] and the lowest well-being across the 27 European Union member states [27]. In the same period, 25.9% of Greek young adults (University students) have shown increased depressive symptoms corresponding to a threshold for clinical depression [28]. Despite these dramatic changes, the prevalence of well-being, the level of physical (in) activity, the status of eating behaviors and the predictive well-being effects of physical (in) activity and eating behaviors have not been explored in Greek adolescents during a COVID-19 lockdown period. This exploration is essential, as many Greek adolescents in the pre-COVID-19 era showed poor eating behavior [29] and of the lowest well-being levels across 31 countries [30]. This exploration should thus be prioritised to update decision-makers on effective policies for adolescents during a lockdown period, especially since 35% of the Greek families reported well-being decreases for their kids (<18 years) in the first lockdown during spring 2020 [31].

Therefore, this study aimed to explore the prevalence of poor well-being, and the levels and predictive effects of physical activity and eating behavior on well-being in adolescents during the second total lockdown period (November 2020–January 2021) in Greece.

## 2. Materials and Methods

The study was conducted in accordance with the Declaration of Helsinki and received ethical approval from the University of Thessaly ethics committee (Re: 1723;/09-12-2020). An online survey was prepared using Google Forms, which allowed gathering information during the COVID-19 pandemic lockdown period. Inclusion criteria for participating in this study were age, 12–17 years, and educational level, attending the secondary Greek education (Gymnasium or Lyceum). First, a social media search was conducted with the aim to allocate groups of interest for parents (schools, institutions, parent/guardian groups or associations). The survey was subsequently communicated to such lists. In this communication parents were informed regarding the aim of the survey, the procedures, and the expected benefits. They were also reassured that participation was voluntary and they, or their children, could withdraw from the completion of the survey if they wished. Parents were subsequently asked to provide their consent for their children’s participation, and finally asked to forward the survey to their children. The completion of the survey took approximately 15 min and was based on self-reports. Data were collected between mid-January and the beginning of February 2021. Hence, the data captures the reactions of participants in the period of the second lockdown period, when school classes were conducted online and the vast majority of sport activities for our target group were interrupted.

### 2.1. Sample

Participants were 950 adolescents (518 boys) aged 12 to 17 years (Mage = 14.41, SD = 1.63), who attended secondary education in Greece (46% junior high school (Gymnasium), 54% high school (Lyceum)). Based on self-reports on weight and high, the mean Body Mass Index (BMI) of participants was 21.06 (SD = 2.30). Among them, 698 (73%) were members of sport clubs with mean sport experience 7.17 (SD = 2.89) years, mean competitive experience 4.70 (SD = 2.26) years; they were training between 3 and 6 days per week (M = 4.57, SD = 1.43 days) for an average of 6.94 (SD = 4.28) hours.

### 2.2. Measures

#### 2.2.1. Physical Activity and Sedentary Behavior

The International Physical Activity Questionnaire Short Form (IPAQ-SF) [32], in particular, the Greek version previously implemented to adolescents [33], was used to assess participants’ physical activity and sedentary behavior. The questionnaire assesses frequency (days/week) and duration (minutes/day) of light, moderate, and vigorous intensity physical activity, and in addition sedentary time per day. The amount of physical activity per week, per category of intensity, is calculated by multiplying the number of days/week reported by the minutes/day reported. Sedentary time is calculated by multiplying the average sedentary time per day by seven. The total physical activity per week was calculated by adding the minutes of the different intensities. Two items similar to the ones of the IPAQ-SF were added in this survey to better capture physical activity during the lockdown period; these asked participants to indicate frequency and duration of physical activity in the house and out of the house (e.g., street, park, sport venues). The scores for these additional items were calculated accordingly.

#### 2.2.2. Mood

The Greek version of the 4-Dimensional Mood Scale (4DMS) [34] was used to assess participants’ mood. The scale consists of 20 adjectives assessing positive energy (positive affectivity and high activation—four items), relaxation (positive affectivity and low activation—five items), negative arousal (negative affectivity and high activation—six items), and tiredness (negative affectivity and low activation—five items). Participants rated each adjective on the extent to which it generally described their mood during the previous seven days using a 5-point Likert format (1 = not at all, 5 = very much).

#### 2.2.3. Psychological Well-Being

The World Health Organization Well-Being Index-5 [33] was used to assess participants’ psychological well-being. This scale consists of five questions focusing on subjective quality of life based on positive mood (good spirits, relaxation), vitality (being active and waking up fresh and rested) and general interest (being interested in things). Participants were asked to indicate their levels of well-being during the previous seven days. Responses were given on a 6-point Likert scale (0 = at no time, 5 = all the time), with lower scores indicating worse well-being. According to the WHO, the Well-Being Index-5 has been translated in more than 30 languages including the Greek language and can be employed for children aged 9 years old and above [33].

#### 2.2.4. Eating Behavior

A slightly modified version of the Short Diet Behaviour Questionnaire for Lockdowns (SDBQ-L) [35,36] was used to address eating behavior for the study population. Following a short description of what consists healthy eating, participants asked to indicate whether (a) they were following a healthy diet, (b) they were eating more than usual, (c) they were eating on a consistent schedule, and (d) they were eating out of control. Responses were provided on a 7-point Likert scale (1 = definitely no, 7 = definitely yes), with higher scores indicating better eating behavior after reversing scores for items ‘b’ and ‘d’. The SDBQ-L has been previously administrated in Greek populations [35].

#### 2.2.5. Data Analysis

Descriptive statistics, Cronbach’s alpha coefficients for the scales, and correlations between all variables are presented in Table 1. The reliability of all scales was satisfactory, ranging from 0.80 to 0.87. Notably, first and foremost, participants’ mean score on well-being index was close to the median point of the scale, below the WHO threshold (<13), indicating a poor well-being [33]. Scores for positive moods were moderate and for negative moods moderate to low. Moderate and vigorous physical activity combined were below the 50% of the recommended by WHO physical activity. Finally, scores on healthy eating habits were moderate. Well-being was strongly, positively related to positive moods and negatively to negative moods. Physical activity and healthy eating habits showed moderate positive correlations with well-being and positive moods, and negative low correlations with negative moods. Finally, sedentary behavior showed negative correlations with well-being and positive moods and positive correlations with negative moods.

## 3. Results

### 3.1. Preliminary Analyses

Descriptive statistics, Cronbach’s alpha coefficients for the scales and correlations between all variables are presented in Table 1. The reliability of all scales was satisfactory, ranging from 0.80 to 0.87. Notably, first and foremost, participants’ mean score on well-being index was close to the median point of the scale, below the WHO threshold, indicating a poor well-being. Scores for positive moods were moderate and for negative moods moderate to low. Moderate and vigorous physical activity combined were below the 50% of the recommended by WHO physical activity for adolescents. Finally, scores on healthy eating habits were moderate. Well-being was strongly, positively related to positive moods and negatively to negative moods. Physical activity and healthy eating habits showed moderate positive correlations with well-being and positive moods, and negative low correlations with negative moods. Finally, sedentary behavior showed negative correlations with well-being and positive moods and positive correlations with negative moods.

### 3.2. Group Differences

Two, two-way (2 × 2) multivariate analyses of variance, one for the psychometric variables (well-being and mood) and one for the behavioral variables (total physical activity, sedentary time, and healthy eating habits), were conducted to test for differences as a function of sex and sport participation. Mean scores for all subgroups are presented in Table 2.

Regarding the psychometric variables, the analyses showed significant multivariate effects for sex, F (5, 941) = 22.21, *p* < 0.01, partial η2 = 0.11, and sport participation, F (5, 941) = 4.87, *p* < 0.01, partial η2 = 0.03, and a non-significant interaction effect, F (5, 941) = 1.45, *p* = 0.20. Examination of the univariate effects for sex revealed significant differences in well-being, F (1, 949) = 74.99, *p* < 0.01, partial η2 = 0.07, positive energy, F (1, 949) = 29.68, *p* < 0.01, partial η2 = 0.03, relaxation, F (1, 949) = 55.76, *p* < 0.01, partial η2 = 0.06, negative activation, F (1, 949) = 66.87, *p* < 0.01, partial η2 = 0.07, and tiredness, F (1, 949) = 18.52, *p* < 0.01, partial η2 = 0.02, with boys scoring higher than girls on well-being and positive moods, and lower on negative moods. Examination of the univariate effects for sport participation revealed significant differences in well-being, F (1, 949) = 18.17, *p* < 0.01, partial η2 = 0.02, and positive energy, F (1, 949) = 11.40, *p* < 0.01, partial η2 = 0.01, with athletes scoring higher than non-athletes, and non-significant effects for relaxation, F (1, 949) = 0.03, *p* = 0.86, negative activation, F (1, 949) = 1.72, *p* = 0.19, and tiredness, F (1, 949) = 1.21, *p* = 0.27.

Regarding the behavioral variables, the analyses showed significant multivariate effects for sex, F (3, 942) = 11.23, *p* < 0.01, partial η2 = 0.04, and sport participation, F (3, 942) = 15.26, *p* < 0.01, partial η2 = 0.05, and a non-significant interaction effect, F (3, 942) = 0.78, *p* = 0.51. Examination of the univariate effects for sex revealed significant differences in total physical activity, F (1, 948) = 21.56, *p* < 0.01, partial η2 = 0.02, and sedentary time, F (3, 942) = 9.46, *p* < 0.01, partial η2 = 0.01, with boys scoring higher in physical activity and lower on sedentary time, and a non-significant effect on healthy eating habits, F (1, 948) = 1.66, *p* = 0.20. Examination of the univariate effects for sport participation revealed significant differences in physical activity, F (1, 948) = 43.95, *p* < 0.01, partial η2 = 0.04, and healthy eating habits, F (1, 948) = 3.89, *p* < 0.01, partial η2 = 0.01, with athletes scoring higher than non-athletes, and a non-significant effect on sedentary time, F (1, 948) = 3.54, *p* = 0.06.

### 3.3. Prediction of Well-Being

Three sets of hierarchical regressions analyses were performed to examine the predictive strength of the behavioral variables for well-being, and the specifics of physical activity in predicting well-being. In all analyses, sex and BMI were entered in the first step to account for sex differences and the effect of BMI on well-being, which were identified in the preliminary analyses.

In the first hierarchical analysis, total physical activity, sedentary time and healthy eating habits were entered in the second step of the analysis. In the first step of the analysis, sex and BMI predicted 13% of the well-being variance, with both predictors being significant (b = −0.34, *p* < 0.01 and b = −0.15, *p* < 0.01, respectively). In the second step, the introduction of the behavioral variables raised the prediction to 27.5% of the variance. All predictors were significant; among the behavior variables, total physical activity was the stronger (b = 0.24, *p* < 0.01), followed by eating habits (b = 0.19, *p* < 0.01) and sedentary time (b = −0.15, *p* < 0.01). The results of this regression analysis are presented in Table 3.

The purpose of the second regression was to identify (a) the role of physical activity intensity and (b) whether frequency (i.e., number of days/week) or duration (total number of minutes/week) of physical activity were stronger predictors of well-being. Thus, in this hierarchical analysis, days and total time of light, moderate and vigorous physical activity were entered in the second step of the analysis. In the second step of the model, the introduction of the physical activity raised the prediction from 13.1% to 23.4% of the variance. Interestingly, the frequency of light, moderate and vigorous physical activity were all significant predictors of well-being, while the duration was not significant for any of them. Similar coefficients were revealed for all types of activity; for vigorous, b = 0.12, *p* < 0.01, for moderate, (b = 0.10, *p* < 0.01), and for light, (b = 0.14, *p* < 0.01). The results of this regression analysis are presented in Table 4.

The purpose of the third regression analysis was to identify the role of physical activity location. Thus, in this hierarchical analysis, days of in-house physical activity and days of out-of-house physical activity were entered in the second step of the analysis. In the second step, the introduction of the physical activity raised the prediction from 13.1% to 22.6% of the variance. Both variables were significant predictors of well-being, with out-of-house physical activity (b = 0.23, *p* < 0.01) being stronger than in-house physical activity (b = 0.17, *p* < 0.01). The results of this regression analysis are presented in Table 5.

## 4. Discussion

The present study aimed primarily at identifying relationships between physical activity, sedentariness, eating behavior and well-being among adolescents during a lockdown period, while school classes were delivered on-line and organized sport activities were interrupted. The most striking result was the worryingly low levels of well-being reported by participants. The average score on well-being, and accordingly the score of 49.5% of the participants, was below the threshold (total score < 13) identified by the WHO as requiring inspection for depressive symptoms [33]. Similarly, 48.3% of Australian adolescents in a COVID-19 lockdown showed mental distress corresponding to a probable mental illness [37]. Poor well-being components have also been recorded in Philippines where 27% to 40% of young people aged 12 to 21 years old showed increased levels of anxiety and/or depressive symptoms [38].

The signaling effects of the COVID-19 on Greek adolescents have been identified during the previous lockdown period, in spring 2020. In a study with parents, it was reported that the psychological health of children (<18 years) was considerably affected in 35% of the Greek families [31]. These findings are similar to the findings from Canada where 35.7% of parents were found to be experiencing high levels of anxiety due to COVID-19 [39]. Similarly discouraging findings were found for young people and/or parents in the previous lockdown period in Italy [40,41] or in Greece; for example, University students in Greece showed considerable reduction of mental health, including increased anxiety and depressive symptoms corresponding to a threshold for clinical depression [28].

Encouragingly, physical activity and healthy patterns of eating behavior were positively related with participants’ positive mood dimensions and well-being. Furthermore, adolescents who used to participate in organized sport reported higher levels of physical activity and better well-being compared to those not participating in organized sport, above the threshold suggested by the WHO for better well-being but still low. Despite the interruption of sport due to the restrictions, which should have had a more dramatic impact on athletes’ physical activity and subsequently well-being, athletes managed to maintain a more active lifestyle and better well-being. Various studies have repeatedly related physical activity with better well-being components including lower depression and anxiety symptoms in adolescents [11,12] and adults [4,6]. In light of this repeated positive evidence, the WHO [13] guidelines recommend adolescents perform physical activity of 60 min/day (≥420 min/week) for better mental health. Physical activity of participants in the present study was below the recommended by the WHO levels, nevertheless, was the stronger predictor of well-being, even after controlling for the effects of sex and BMI that were also found to influence well-being. The beneficial effects of physical activity for mental health during the COVID-19 pandemic have been also evidenced in young Chinese persons by the authors of [16], who reported that physical activity for >60 min/week was linked to lower risk of deteriorated well-being.

Healthy eating emerged as a secondary significant predictor of better well-being. Indeed, healthy eating in the COVID-19 pandemic is linked to improved well-being components such as lower depressive and anxious symptoms [42]. However, eating behavioral patterns have deteriorated in the COVID-19 pandemic (e.g., number of main meals or snacks between meals, eating out of control or type of food) [36]. Furthermore, data from Cyprus for the pre- and post-lockdown period due to COVID-19 reported a significant increase of young people (5–14 years old) consuming food items containing sugar [18]. Initiatives towards healthier eating behavior at home are thus essential, and relevant updates have been recently published for further action. In particular, the importance of addressing healthy dietary behavioral patterns represents a constant concern. State-of-the-art outlines have reported an update for healthy eating across five continents and for Mediterranean diet, with physical activity being an inherent component [22,23]. Such initiatives, however, seem to be potentially challenging due to adolescents’ poor well-being. Despite this somber reality, the multiple benefits of physical activity reveal promising perspectives towards healthier eating. In particular, physical activity lowers sedentariness and sleep disturbance [19], which are, in turn, linked to less deteriorated dietary behavior [20,21]. This beneficial sequence seems to be supported, although not examined, in our study by the correlations amongst house-based physical activity, better eating behavior and improved well-being.

In contrast to physical activity and healthy eating behavior, sedentary time predicted well-being deterioration in our study. This is an unsurprising finding because sedentary time has been repeatedly associated with deteriorated well-being components (e.g., depression) in large-scale epidemiological studies across adolescents [7,8] and adult populations [3]. Moreover, adolescents living in COVID-19 lockdowns have demonstrated increased sedentariness and low well-being [14,15,16,17,18,19]; sitting time in particular has dramatically increased [36] and clearly related with deteriorated well-being components including sleeping patterns and depressive and anxiety symptoms [43]. Tackling sedentary time in Greek adolescents during the COVID-19 pandemic and involved lockdowns should thus be prioritized to decrease sitting time modalities such as the widespread screen time. Towards this direction, it would be interesting to consider the potentially detrimental effects of online schooling on physical activity, and address to school directors and policy makers the importance of physically active breaks between classes in the daily schedule.

A final issue deserving consideration is the relevant contribution of physical activity modalities towards the prediction of well-being. The first notable point is that frequency of physical activity (days/week) was a stronger predictor of duration (total minutes/week), for all physical activity intensity modes. The emerged importance of frequency in physical activity participation has significant implications for how the recommendations for physical activity that should be prioritized, at least during the pandemic times. Small bouts of physical activity may increase overall frequency (days/week) without increasing risks of injuries, which are typically linked to accumulated physical activity volume. In line with this, a recent study has reported the contribution of small bouts to increased frequency of physical activity among children [44]. The second issue is that out-of-house physical activity was a stronger predictor of well-being; yet, after accounting for that effect, in-house physical activity was still a significant predictor. Even though the superiority of the out-of-house activity is a reasonable finding, that in-house physical activity during a lockdown time, where the chances of going out of the house are limited, highlights the unique impact that physical activity per se, not combined with going out, can have on well-being. Therefore, the promotion of ideas and innovative ways to exercise in the house should be particularly emphasized during lockdown periods.

## 5. Limitations

The findings of the current study should be considered under the light of certain limitations. First, the cross-sectional design of the study does not allow causal conclusions with regard to the statistical prediction of well-being from physical activity, sedentariness and eating habits; second, the reliance on self-reported behavioral measures, including physical activity, sedentary behavior and eating habits, which are dependent on memory and, in cases, liable to social desirability; third, the consideration of additional, to gender and BMI, control variables that may have influenced the results; and finally the recruitment of the sample through the social media, which does not allow calculation of response rates and limits the participation of people without access to the internet or non-users of social media, thus limiting the generalizability of the findings. These limitations, despite being typical in large scale surveys, and in particular web-based surveys, suggest that our findings should be interpreted with caution.

## 6. Conclusions

Action needs to be taken by both researchers and policy makers to enhance well-being in Greek adolescents by providing guidance and opportunities for increased physical activity, decreased sedentary time and healthier eating behaviors. Such action is of high priority as 50% of our sample showed levels of well-being below the general population norms. These levels signify according to the WHO the need to subsequently investigate for potential depression; thus, prompt research is warranted to address this important health risk that emerged during the pandemic. Considering evidence suggesting that Greek adolescents’ physical activity and well-being are among the lowest in Europe even before the emergence of the pandemic [30], initiatives targeting the improvement of healthy habits should be supported hereafter with consistency.

## Figures and Tables

**Table 1:** Descriptive statistics, Cronbach’s alpha, and correlations.

	Descriptives		Alpha	Correlations									
	M	SD		1	2	3	4	5	6	7	8	9	10
1. Well-being	12.63	5.92	0.84	-									
2. Positive energy	3.00	0.98	0.87	0.67 **	-								
3. Relaxation	2.75	0.81	0.80	53 **	0.46 **	-							
4. Negative activation	2.07	0.89	0.87	−0.45 **	−0.25 **	−0.35 **	-						
5. Tiredness	2.03	0.80	0.85	−0.33 **	−0.17 **	−0.19 **	0.51 **	-					
6. Eating behaviour	4.45	1.21	0.75	0.24 **	0.29 **	0.13 **	−0.09 **	−0.09 **	-				
7. Light PA	247.78	209.30	-	0.28 **	0.30 **	0.11 **	−0.07 *	0.00	0.11 **	-			
8. Moderate PA	97.28	119.55	-	0.24 **	0.22 **	0.08 **	−0.07 *	−0.04	0.13 **	0.39 **	-		
9. Vigorous PA	70.23	104.06	-	0.27 **	0.29 **	0.06	−0.07 *	0.01	0.14 **	0.35 **	0.31 **	-	
10. Total PA	415.28	334.29	-	0.35 **	0.36 **	0.12 **	−0.10 **	−0.01	0.16 **	0.87 **	0.70 **	0.64 **	-
11. Sedentary time	439.11	216.57	-	−0.24 **	−0.27 **	−0.10 **	0.19 **	0.14 **	−0.10 **	−0.15 **	−0.03	−0.11 **	−0.14 **

* p < 0.5, ** *p* < 0.01; M: Mean; SD: Standard Deviation.

**Table 2:** Mean scores for all subgroups.

	Boys		Girls		Athletes		Non-Athletes	
	M	SD	M	SD	M	SD	M	SD
1. Well-being	14.41	5.55	10.47	5.63	13.22	5.90	10.96	5.65
2. Positive energy	3.22	0.95	2.73	0.96	3.07	1.00	2.86	0.91
3. Relaxation	2.95	0.76	2.50	0.79	2.77	0.83	2.70	0.75
4. Negative activation	1.83	0.72	2.36	0.97	2.03	0.84	2.18	0.99
5. Tiredness	1.93	0.71	2.16	0.89	2.01	0.77	2.11	0.89
6. Eating behaviour	4.38	1.18	4.53	1.24	4.49	1.23	4.34	1.14
7. Light PA	281,96	221,42	205,71	184,62	267,57	209,79	190,99	196,03
8 Moderate PA	106,74	123,19	85,80	114,24	112,57	128,05	54,58	77,79
9. Vigorous PA	85,42	118,82	51,74	79,26	80,40	108,28	41,51	85,16
10. Total PA	474.12	359.28	343.25	288.00	460.55	339.34	287.09	281.84
11. Sedentary time	413.73	210.68	469.55	219.82	429.29	211.53	466.37	228.19

M: Mean; SD: Standard Deviation; PA: Physical Activity.

**Table 3:** Prediction of well-being from behavioral variables.

	Beta	t	R^2^	F
step 1			13	(2, 914) = 68.40 **
Sex	−0.33	−10.95 **		
BMI	−0.15	−4.89 **		
step 2			27.5	(5, 914) = 68.96 **
Sex	−0.27	−9.54 **		
BMI	−0.09	−3.28 **		
Total PA	0.24	8.03 **		
Sedentary time	−0.16	−5.49 **		
Eating behavior	0.19	6.57 **		

** *p* < 0.01; BMI: Body Mass Index; PA: Physical Activity.

**Table 4:** Prediction of well-being from physical activity intensity, frequency and duration.

	Beta	t	R^2^	F
step 1			13.1	(2, 915) = 68.88 **
Sex	−0.33	−10.95 **		
BMI	−0.15	−4.89 **		
step 2			23.4	(8, 914) = 34.55 **
Sex	−0.26	−8.80 **		
BMI	−0.10	−3.57 **		
Light PA-days	0.13	4.01 **		
Light PA-time	0.03	0.92		
Moderate PA-days	0.10	2.55 *		
Moderate PA-time	0.04	1.25		
Vigorous PA-days	0.12	2.87 **		
Vigorous PA-time	0.01	0.36		

* *p* < 05; ** *p* < 0.01; BMI: Body Mass Index; PA: Physical Activity.

**Table 5:** Prediction of well-being from physical activity location.

	Beta	t	R^2^	F
step 1			13.1	(2, 913) = 68.89
Sex	−0.34	10.99 **		
BMI	−0.15	4.92 **		
step 2			22.6	(4, 911) = 66.43
Sex	−0.27	9.10 **		
BMI	−0.11	3.79 **		
Home PA	0.17	5.59 **		
Out-of-home PA	0.23	7.41 **		

** *p* < 0.01; BMI: Body Mass Index; PA: Physical Activity.

## Data Availability

Data are available upon request from the first author.
